# Fabrication Principles and Their Contribution to the Superior In Vivo Therapeutic Efficacy of Nano-Liposomes Remote Loaded with Glucocorticoids

**DOI:** 10.1371/journal.pone.0025721

**Published:** 2011-10-06

**Authors:** Yuval Avnir, Keren Turjeman, Deborah Tulchinsky, Alex Sigal, Pablo Kizelsztein, Dina Tzemach, Alberto Gabizon, Yechezkel Barenholz

**Affiliations:** 1 Laboratory of Membrane and Liposome Research, Department of Biochemistry, The Hebrew University-Hadassah Medical School, Jerusalem, Israel; 2 Laboratory of Experimental Oncology, Shaare Zedek Medical Center, Jerusalem, Israel; Aristotle University of Thessaloniki, Greece

## Abstract

We report here the design, development and performance of a novel formulation of liposome- encapsulated glucocorticoids (GCs). A highly efficient (>90%) and stable GC encapsulation was obtained based on a transmembrane calcium acetate gradient driving the active accumulation of an amphipathic weak acid GC pro-drug into the intraliposome aqueous compartment, where it forms a GC-calcium precipitate. We demonstrate fabrication principles that derive from the physicochemical properties of the GC and the liposomal lipids, which play a crucial role in GC release rate and kinetics. These principles allow fabrication of formulations that exhibit either a fast, second-order (t_1/2_ ∼1 h), or a slow, zero-order release rate (t_1/2_ ∼ 50 h) kinetics. A high therapeutic efficacy was found in murine models of experimental autoimmune encephalomyelitis (EAE) and hematological malignancies.

## Introduction

Glucocorticoids (GCs) are widely used drugs in treatment of inflammatory diseases and cancer [1]–[3]. However, treatments based on GCs are routinely accompanied by adverse effects related to their unfavorable pharmacodynamics, unfavorable pharmacokinetics, and biodistribution. When GCs are given intravenously or orally, their pharmacokinetics obey first-order release kinetics [4] with an initial high plasma concentration followed by rapid exponential clearance. This unfavorable pharmacokinetics can be overcome by the use of a drug delivery system (DDS) providing a slow, zero-order release kinetics that, while keeping plasma drug concentration below toxicity level, will allow for the drug concentration needed to achieve a therapeutic effect [5]. Such performance can be obtained by using several different drug delivery approaches including: transdermal pumps [6] and xenotransplantation [7], or different types of injectable nano-particulates (nano-DDS) including polymers [8], and liposomes [9]. Injectable nano-DDS are advantageous as they utilize the unique micro-anatomy of the inflamed tissue blood capillaries, which have gaps between the lining endothelial cells causing vessel leakiness. Inflamed and cancerous tissues can discriminate between nano- and micro-particles, as only the nano-particles can extravasate into these tissues. This size-dependent “passive targeting” to the inflamed and cancerous tissues [10]–[12] is the Achilles heel of the disease permitting better treatment of inflammation and cancer.

Using liposomes encapsulating GCs for treatment of inflammatory diseases was one of the first targets of “Liposomology” and remained a major goal for over four decades [12], [13]. Research on liposomes encapsulating GCs for cancer treatment was also performed, however to a much lesser extent [14], [15]. Despite the progress achieved, the challenge of making a stable and efficacious GC-loaded nanoliposome formulation having slow zero-order release kinetics was not met.

In order to address this challenge we decided to apply the strategy of transmembrane ion gradient-driven remote loading of amphipathic weak acid or base drugs into pegylated nano-liposomes (nano-sterically stabilized liposomes, referred to as nSSL). Historically, in our laboratory the remote loading approach began from the need to achieve a highly efficient and stable liposomal drug encapsulation that would be stable during storage and have a long circulation time in the blood. This approach, which was recently discussed by us in detail for 9 different drugs [16], was applied for remote loading of the amphipathic weak base anticancer drug doxorubicin [16]–[20] and was the basis for the first FDA-approved pegylated nano-liposomal anticancer drug, Doxil™.

Since the approval of Doxil™ (1995), this approach was successfully employed for additional drugs and agents, in our laboratory and in those of others [16], [21]–[24].

Overall, from our own experience, we concluded that a transmembrane ammonium sulfate gradient is most suitable for stable encapsulation of amphipathic weak bases [16]–[19], [25], [26], while a transmembrane calcium acetate gradient is most suitable for loading amphipathic weak acids [16], [27], [28].

The theoretical basis of these nanochemical engines, described elsewhere [9], [18], [20], is summarized in **Scheme S1**, (Supporting information). The core technology of this approach is that liposomes are fabricated to exhibit the desired pH and/or ion gradient by their encapsulation with salts composed of either weak bases (e.g., ammonium) [16], [17] or weak acids (e.g., acetate) [16], [28]. The degree of ionization of these compounds is pH dependent, and their ionized species (i.e., ammonium and acetate) have a very low permeability coefficient and octanol-to-buffer partition coefficient; therefore they either do not, or only very slowly, permeate the liposome lipid bilayer, while their un-ionized species have high permeability, as well as octanol-to-buffer partition coefficient (exemplified by ammonia and acetic acid) and therefore can diffuse relatively fast across the lipid bilayer and reach the intraliposome aqueous phase [16], [20]. The magnitude of the intraliposome high/external medium low transmembrane gradient of such ions is the driving force for remote loading, as they can be exchanged with amphipathic drugs that are weak acids or bases. The counterion of the gradient-forming ion (e.g. sulfate or calcium in the case of ammonium or acetate gradient, respectively) can be selected so that it will control the state of aggregation and precipitation/crystallization of the drug–counterion salt in the intraliposome aqueous phase, thereby contributing to control efficiency and stability of remote loading, as well as drug release rate at various temperatures [16], [18]–[20], [26], [28].

It is important to note that the successful application of this nanochemical engine benefits from the very small trapped aqueous volume of nanoliposomes (2.21×10^5^ nm^3^ for a 37.5 nm radius liposome), which supports faster and higher accumulation and intraliposome precipitation of drug–counterion salt in crystalline or noncrystalline forms.

Recently, we reported success in using these GC-loaded nanoliposomes (nSSL-GC) for treatment of an adjuvant- induced arthritis model in rats [27]. The GCs used there are the amphipathic weak acid GC pro-drugs methylprednisolone hemisuccinate sodium salt (MPS) and betamethasone hemisuccinate sodium salt (BMS). nSSL-GC based on high (>37°C) T_m_ “liposome-forming phosphatidylcholines” are characterized by the unique property of being a zero-order, slow drug release DDS, as shown *in vitro* in human plasma and inflamed synovial fluid, and *in vivo* in the rat's systemic circulation and inflamed paw [27].

This study focuses on the relevance of the physicochemical aspects of these nSSL-GC to their superior therapeutic performance and it complements our 1995 publication [28], which describes some basic principles of the remote loading of amphipathic weak acids using model molecules *in vitro*.

## Results and Discussion

### Amphipathic weak acid glucocorticoid prodrugs are optimal candidates for nSSL remote loading

Most approaches of remote drug loading into liposomes require the loaded drug to be an amphipathic weak base [9], [16], [18] or acid [16], [27], [28]. An *in silico* database screening of suitable commercially available GC candidates for remote loading yielded only water-soluble weak acid GCs: the GC-succinates (GC-S) and the GC-Phosphates (GC-P). No GCs that are amphipathic weak bases were found. Therefore, we chose to study the remote loading approach driven by the intraliposome-high/extraliposome medium-low calcium acetate transmembrane gradient previously described by us for the loading of amphipathic weak acids [28] (described in **Scheme S1a**, Supporting information).

Efficient remote loading of GCs requires the GC candidate to have a specific set of physicochemical features. Firstly, the GC should be fairly water-soluble, and secondly, while present in the extraliposomal medium, a part of the GC molecule has to be hydrophobic enough to allow sufficient GC diffusion across the liposome lipid bilayer in its uncharged form (see **Scheme S1b**, Supporting information), while inside the intraliposome aqueous phase the hydrophobicity/hydrophilicity ratio should be reduced so that rate of release of GC from the intraliposome aqueous phase into the external medium will be slowed down. This is achieved firstly by ionization of the loaded GC, which is enhanced by precipitation of the loaded GC in the intraliposome aqueous phase as an insoluble Ca(GC)_2_ salt. This precipitation is related to the steroid ring structure and to the nano-intraliposomal aqueous volume of the nSSL. Another important condition for a successful formulation is having a high “energy of activation”, which is the slope of the Arrhenius plot [17] that describes the rate of GC release from nano-liposomes as a function of the reciprocal of the Kelvin temperature (1/T). Such a high slope enables a sufficient release at 37°C (needed to achieve efficacy), concomitant with a minimal release at 2–8°C (to ensure long-term storage shelf life), both of which were demonstrated for Doxil [17], [19].

**Figure 1:**
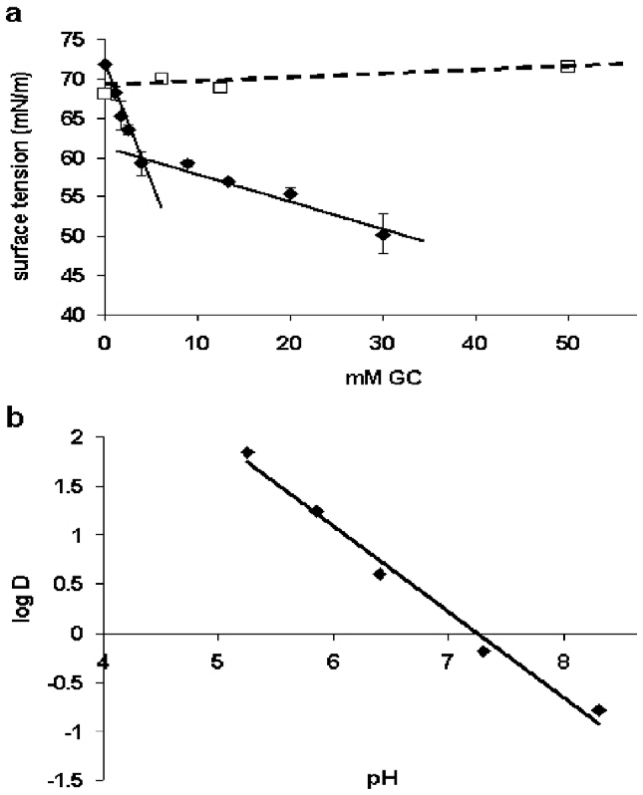
MPS physicochemical parameters relevant to GC remote loading. (**a**)Comparing surface tension of MPS (♦) and DEXP (□) as a function of GC concentration. (**b**) Octanol/buffer log distribution of MPS (using 20 mM HEPES at the specified pH as buffer).

Both GC acid prodrug families, the succinate and the phosphate sodium salts, meet the conditions of sufficient water solubility. However, only the GC-S, represented by MPS, but not the GC-P ,represented by dexamethasone phosphate (DEXP), were shown to be surface-active, as was demonstrated by the reduction of surface tension of MPS, but not DEXP (**Fig. 1a**). In addition, the fact that for MPS, surface activity dependence on its concentration is characterized by a biphasic curve indicates that it undergoes self-association at a critical aggregation concentration (CAC) [29], [30], which is ∼4.3 mM (**Fig. 1a**). The amphipathic weak-acid characteristic is further supported by the linear pH-dependency of logD for the pH range of 5–8.5 (**Fig. 1b**). At pH 5 the logD is 2 and at pH 8.5 the logD is -0.75. DEXP shows no partitioning to the octanol phase throughout this pH range.

**Figure 2:**
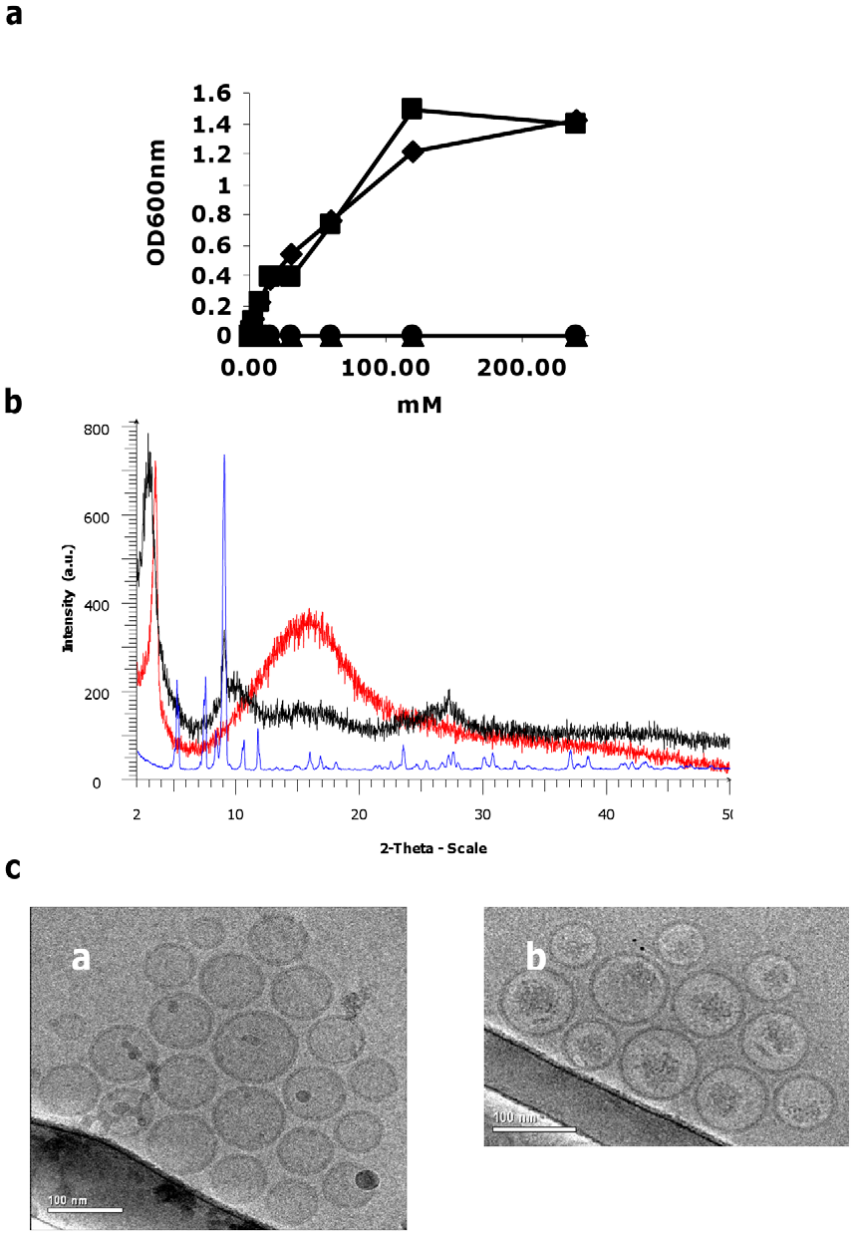
Calcium-MPS interaction studies. (**a**) Change of turbidity (OD at 600 nm) of 20 mM MPS solution at pH 7.4 generated by mixing MPS with different salt solutions: calcium acetate (♦), calcium chloride (▪), sodium acetate (▴), and sodium chloride (•). (**b**) X-ray diffraction pattern of calcium acetate (blue), MPS (red) and calcium-MPS (black) powders. (**c**) Cryo-TEM images of nSSL (white **a**) before and (white **b**) after MPS loading (reprinted with permission from *Langmuir* [31] and *Arthritis and Rheumatism* [27]).

**Fig. 2a** demonstrates that calcium ions (but not sodium ions) cause MPS aggregation, whereby characterization of the precipitate by XRD analysis shows a clear crystalline nature of Ca(MPS)_2_ (**Fig. 2b**). Thus, we concluded that GC-S are highly favorable candidates for remote loading via the calcium acetate transmembrane gradient, while the GC-P are not. This prediction was supported by the highly efficient loading of MPS compared with the poor remote loading of betamethasone phosphate (BMP) (see details below).

### Understanding the mechanism of MPS remote loading

**Figure 3:**
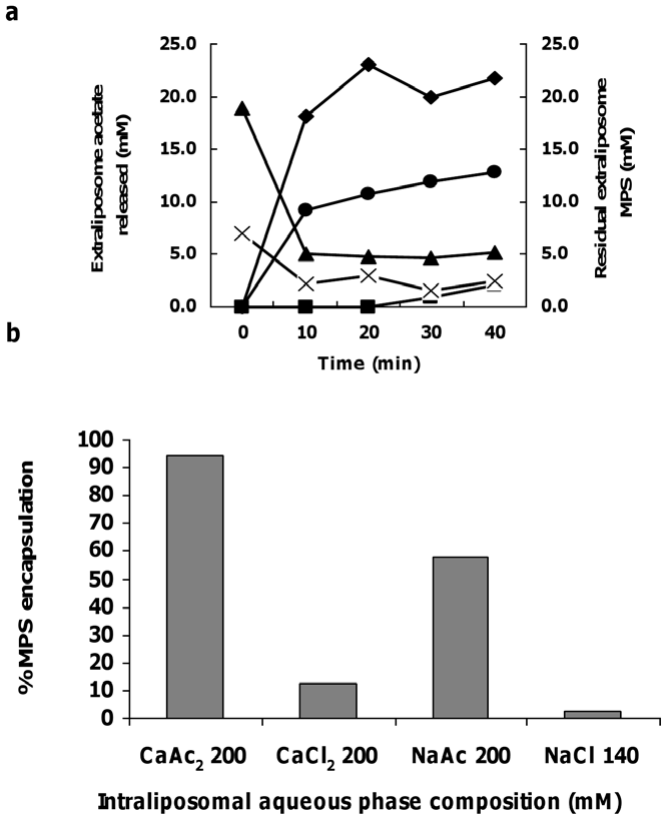
Studying the kinetics of nSSL acetate release and MPS encapsulation during nSSL remote loading, and the effect of intraliposome salt composition on MPS loading. (a)Reduction in level of extraliposome MPS during remote loading of 20 mM (▴) and 6.66 mM MPS (×). Extraliposome acetate increase induced by remote loading of 6.66 mM MPS (•) and 20 mM MPS (♦). As a control, acetate release from nSSL with no MPS present in the bulk medium (▪). (b) Effect of intraliposome salt composition on MPS remote loading.

In order to demonstrate that MPS is remote loaded by the mechanism proposed in **Scheme S1** (Supporting information) and in order to optimize this procedure we conducted the following set of experiments:

(**I**) We characterized the remote loading mechanism of MPS by studying in parallel the kinetics of MPS influx (out/in) and acetic acid and calcium ions efflux (in/out) of the nSSL respectively. **Fig. 3a** shows that most of the remote loading occurs during the first 15 min of incubation, and this encapsulation is paralleled by an efflux of an almost stoichiometric (∼mole per mole) amount of acetic acid from the nSSL (both molecules when fully dissociated have a singly charged ion). (**II**) In addition, previously we have published cryo-transmission electron microscopy (Cryo-TEM) images of nSSL remote loaded with MPS (nSSL-MPS) and nSSL [27], [31] (**Fig. 2c**), and the nSSL-MPS image indicated that MPS uptake into the nSSL leads to the appearance of a precipitate in the intraliposome aqueous phase. The XRD studies conducted here only strengthen the probability that a crystalline calcium-MPS complex is formed (probably Ca(MPS)_2_).

(**III**) In order to prove the advantages of using a calcium acetate transmembrane gradient for MPS loading it was compared with other salts. For this, nSSL (of identical lipid composition) were hydrated with the following salt solutions: sodium chloride, sodium acetate, calcium chloride, and calcium acetate. After the hydration step, nSSL were prepared and remote loaded with MPS as described in Methods. **Fig. 3b** clearly shows that for efficient loading there is a need for both the acetate anions and the calcium cations. This stems from the findings that nSSL hydrated in either calcium acetate or sodium acetate show a much better loading efficiency than nSSL hydrated in calcium chloride or sodium chloride, signifying the necessity of the acetate anion, while calcium as counterion improves MPS loading more than sodium as counterion as is proven from comparing nSSL prepared from lipids hydrated in calcium acetate and in sodium acetate. Moreover, it is important to note that calcium (but not sodium) cations induce MPS precipitation (**Fig. 2a**).

Thus, overall (**Figs. 1**–**3** ****) the “heart” of MPS remote loading by nSSL exhibiting a calcium acetate transmembrane gradient is basically an exchange process between MPS and acetate anions, which is followed by precipitation of a calcium MPS salt in the intraliposome aqueous phase. The fact that all calcium ions remain entrapped in the intraliposome aqueous phase during acetate release, MPS loading, and upon storage points to the integrity of the liposome membrane under the conditions that the uncharged acetic acid can diffuse via this membrane.

### Comparison of remote loading of MPS with other water-soluble GCs and with passive loading of MPS

In order to determine if our loading approach is applicable to other GC-S and to GC-P, we compared remote loading of MPS with two GC-S, and one representative of the GC-P, BMP. This comparison included the following GCs: (1) hydrocortisone hemisuccinate (HCS), characterized by a logD  =  -0.054 at pH 7.0 [32], having one-fifth the anti-inflammatory potency of MPS [33]; (2) betamethasone hemisuccinate (BMS), characterized by a logD  =  -0.01 at pH 7.0 [32], having five times the anti-inflammatory potency of MPS [33]; (3) MPS logD  = 0.02 at pH 7.0 [32]; (4) BMP, characterized by a logD  =  -4.01 at pH 7.0 [32] having five times the anti-inflammatory potency of MPS [33]. As expected, from the logD values, all of the GC-S tried were successfully remote loaded into HSPC/CHOL/2000-PEG-DSPE nSSL with high encapsulation efficiencies: HCS 100%, BMS 80%, and MPS 96%, while the representative of GC-P, BMP, showed minimal encapsulation as explained by its lack of amphipathicity.

We were also interested in comparing remote loading to the passive loading of MPS. This comparison showed a clear advantage of the remote loading method, which achieved 40-fold higher encapsulation efficiency (96% versus 2%) and a 3.2-fold higher intraliposome MPS concentration for the remote loaded nSSL-MPS (38 [GC]/[PL] versus 12[GC]/[PL]).

In addition, the actual MPS concentration in the intraliposome aqueous trapped volume was determined. For this we calculated the actual trapped volume i.e., µl/µmol PL (V_t_). As we proved above and below, the Ca^2+^ ions practically do not leak out of the nSSL during loading and 4°C storage. Therefore, the concentration of entrapped Ca^2+^ was used to determine the trapped volume. A value of 1.2 µl/µmol PL was obtained. Therefore, encapsulation of 14.96 µmol MPS for 34.72 µmol PL calculates into MPS intraliposome concentration of 358 mM, which means that 90% of the nSSL acetate was replaced with MPS.

### Release kinetics as a function of liposome lipid composition and bulk medium composition

Having nSSL with a slow but sufficient zero-order MPS release rate is one of the most important features of a viable nSSL-MPS formulation (see Introduction). Therefore we studied MPS release rate from nSSL-MPS as a function of liposome lipid composition and bulk medium composition at 37°C. This is an expansion of our previous study [27] where we have shown that nSSL-MPS when incubated in human plasma and inflamed synovial fluid results in the controlled release of MPS.

**Figure 4:**
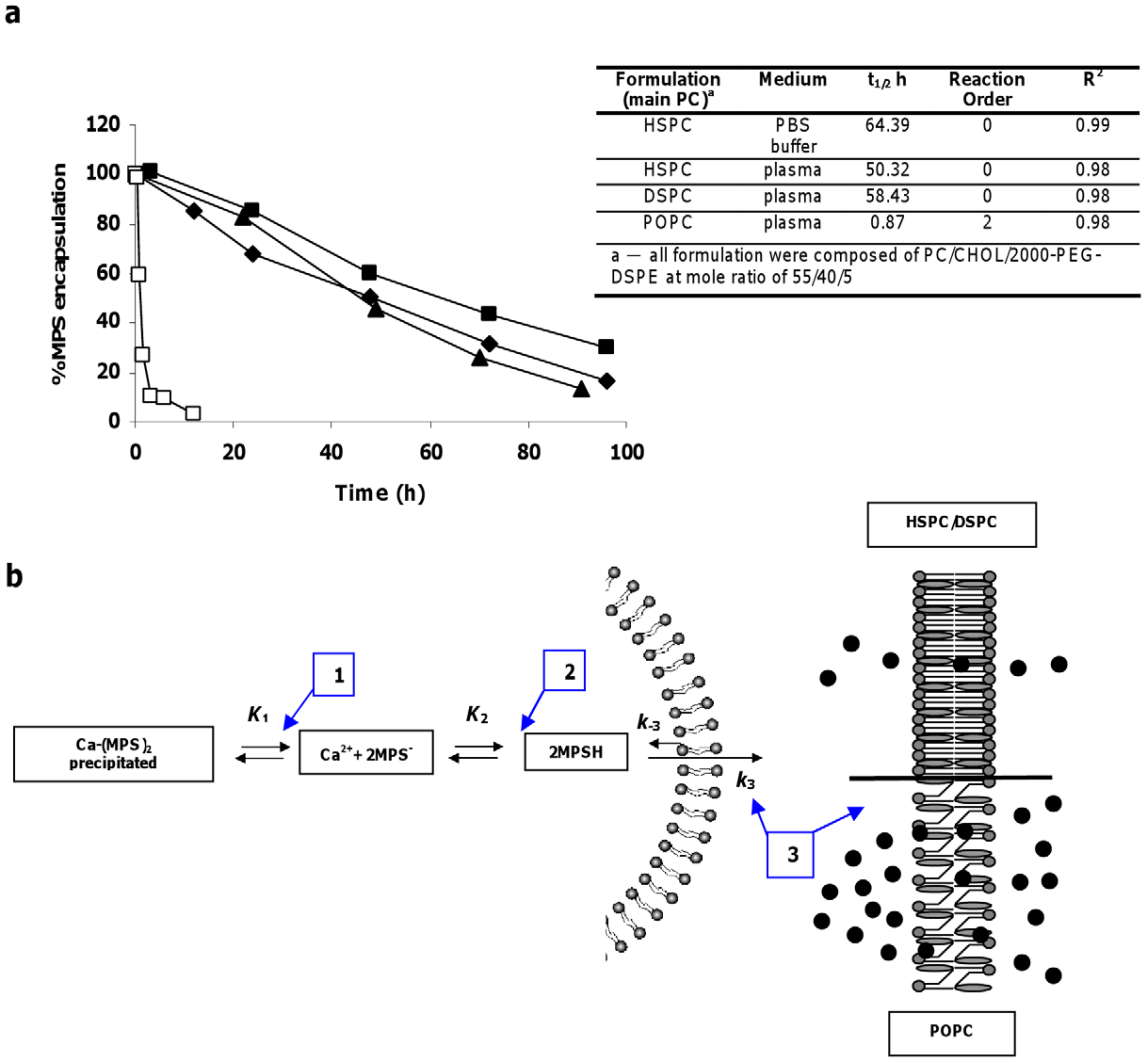
Effect of nSSL lipid compositions and external medium composition on kinetic order and rate of MPS release from nSSL-MPS at 37°C. (**a**) Release of MPS from nSSL composed of: HSPC/CHOL/2000-PEG-DSPE (55/40/5 mole ratio, DSPC being a high T_m_ PC) (▴) (reprinted with permission from Arthritis and Rheumatism [27]);DSPC/CHOL/2000-PEG-DSPE (55/40/5 mole ratio), when incubated at 37°C in plasma (♦); nSSL composed of HSPC/CHOL/2000-PEG-DSPE (55/40/5 mole ratio, HSPC being a high Tm PC), at 37°C in PBS buffer (pH 7.4) (▪); nSSL composed of POPC/CHOL/2000-PEG-DSPE (55/40/5 mole ratio, POPC being a low T_m_ PC) at 37°C in plasma (□). Inset Table — Calculated release kinetic parameters. (b) Scheme describing the mechanism of kinetic order and release rate: 1. In nano-liposomes of all lipid compositions (applies to all liposomes!) used in this study, calcium-MPS salt inside the nano-liposomes is in an intraliposome pH- and temperature-dependent equilibrium (K_1_) between its precipitated salt form, and its soluble dissociated and un-dissociated forms. Since salt solubility increases with rise in temperature from 4°C to 37°C thereby shifting the equilibrium to increased level of the dissociated products. 2. MPS dissociates from its calcium salt is in equilibrium (*K_2_*) between its protonated (uncharged) and unprotonated (charged) form. This equilibrium is dependent on *K_1_*, as the intraliposome pH, is slightly alkaline, only a small fraction MPS is in the protonated neutral form. 3. Protonated (uncharged) MPS can transverses the lipid bilayer in both directions effluxing (*k*_3_) and influxing (*k*_-3_). The net direction of movement is dependent on various conditions including lipid membrane composition, drug physicochemical properties (such as partition coefficient and polar to non-polar surface area, pH, gradient, dilution, motion, mixing and others Zucker et al [16]. For the efflux/influx reactions equilibrium is not reached. The effect of interplay between liposome bilayer lipid composition and the temperature is mediated by bilayer level of “free volume”, as discussed by Stein [34], and reviewed by Barenholz and Cevc [29]. The results described by **Fig 4a** clearly demonstrate that for nSSL based on high-T_m_ PCs (HSPC or DSPC) there is a slow, zero-order kinetics MPS release, while for the nano-liposomes based on the low-T_m_ DOPC, release rate is fast, second-order. This difference agrees well with the fact that the low T_m_-based nSSL has much higher level of liposome membrane free volume (for more in depth discussion on this issue see Results section entitled: “Release kinetics as a function of liposome lipid composition and bulk medium composition”).

The results presented in **Fig. 4a** and its inserted **Table** show the crucial role that nSSL membrane phosphatidylcholine (PC) species has on determining kinetic order and release rate of MPS. nSSL based on high-T_m_ PCs (either HSPC or DSPC) are characterized by similar zero-order release profiles, which translate to similar long t_1/2_ (50 h for HSPC and 58 h for DSPC), and to a constant release rate of ∼1% of the original MPS load per hour, while the low-T_m_ “fluid” POPC-based nSSL showed a much faster release rate that is best described by second-order kinetics, having a t_1/2_ of 0.87 h (almost complete MPS release in less than 2 hours).

**Fig. 4a** and its inserted **Table** also demonstrate that there is no appreciable effect of plasma components on the release rates, which at 37°C are similar in human plasma (t_1/2_ of 50 h) and in PBS (t_1/2_ of ∼64 h). Moreover, as we have reported in our previous study [27], the release of MPS from nSSL based on HSPC/CHOL/2000-PEG-DSPE when incubated in human plasma was neither accompanied by loss of liposome calcium ions nor by change in liposome size distribution (data not shown), indicating that MPS release is not a result of a destructive effect on membrane barrier properties, but rather a controlled release related to the amphipathic nature of the GC.

The large differences in kinetic order and rate of MPS release at 37°C between nSSL based on the fluid, low-T_m_ POPC and the solid, high-T_m_ HSPC or DSPC for a similar level of remote loading of MPS (using the same transmembrane calcium acetate gradient for all 3 formulations) indicate that the nSSL membrane lipid composition is the main factor that determines the release profile of the amphipathic weak acid.

These effects can be explained by the level of liposome membrane “free volume” defects [29], [34], which represent the number of transporting units (**Fig. 4b**) that determines the kinetic order and rate of drug release. In the case of solid, high-T_m_ HSPC or DSPC-based liquid ordered (LO)-phase, the level of free volume defects is low [20], [29] resulting in superior barrier properties and low adiabatic compressibility [35]. Thus, in nSSL based on high-T_m_ PCs the small number of free volume defects becomes saturated already at low MPS concentration and consequently the system operates under conditions of V_max_, leading to a slow, zero-order release. nSSL that are based on the fluid, low-T_m_ POPC, are characterized by lipid bilayers having higher compressibility [35]. Under such conditions the LO phase membrane has a much larger number of free volume defects (equivalent to high V_max_) that can not be saturated by the MPS, and hence release rate does not reach V_max_ and therefore is expected to be fast, and at higher kinetic order, second order (**Fig. 4b**). A simple way to demonstrate the difference in the release order kinetics through the LO membrane is to compare it with the way people escape from a large crowd in a hall under conditions that the number of available gates is limited to the situation that the number of available gates is unlimited. Limited gates are a bottleneck that forces a constant release rate over a long time period, while in a situation of unlimited gates the rate of evacuation can be accelerated by rushing the people through the many gates which, due to their large number, can not be saturated.

Furthermore, for the LO phase-nSSL based on the high-T_m_ HSPC or DSPC liposome-forming lipids, a high activation energy for MPS release (37.8 kcal/mole) was deduced from the large Arrhenius plot slope (describing release rate in the range of 25–55°C as a function of the reciprocal of the Kelvin temperature (1/T). The high activation energy means that it is not easy to increase the number of release sites (gates). This is an important advantage of this formulation, as it ensures nSSL-MPS loading stability upon long term storage at 4°C without compromising the needed slow zero-order release at 37°C. In this respect, nSSL-MPS behaves similarly to Doxil^TM^ [16]–[19].

### Pharmacokinetic and biodistribution advantages of nSSL-MPS

Studies which compare the pharmacokinetics and biodistribution of MPS as nSSL-MPS and free (non-liposomal) drug were performed in the acute EAE mouse model. For these studies nSSL radio-labeled by the non-transferable non-metabolizable ^3^H- cholesteryl hexadecyl ether (CE) as a liposome marker were used in order to follow the fate of both the nano-liposomes and the MPS drug.

**Figure 5:**
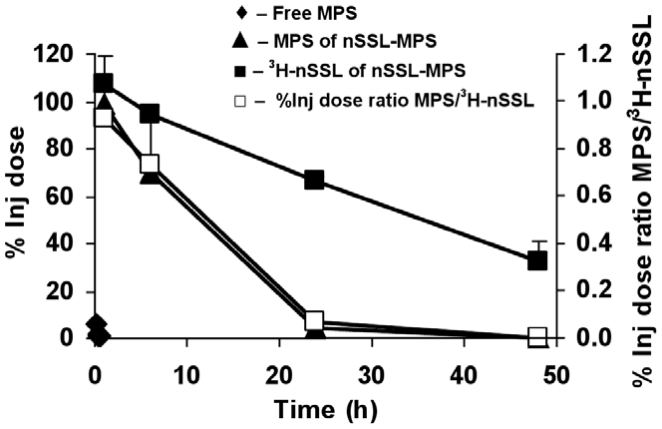
*In vivo* pharmacokinetic study in mice of ^3^H-nSSL-MPS and of free MPS. Pharmacokinetics of free MPS (♦), MPS of nSSL-MPS (▴), ^3^H-nSSL of nSSL-MPS (▪), and the ratio between encapsulated MPS and its carrier ^3^H-nSSL (□).The change in this ratio with time is used as a method to calculate *in vivo* MPS release rate from ^3^H-nSSL-MPS.

**Fig. 5** shows the clear pharmacokinetic advantages that MPS delivered via nSSL has over non-encapsulated (free) MPS. While non-encapsulated MPS is rapidly cleared in minutes (t_1/2_ of ∼9 min), encapsulated MPS is cleared in hours (t_1/2_ of ∼12 h). **Table 1** shows the MPS BD nSSL advantages over free (non-liposomal drug. During the first hour post intravenous (IV) injection, the liver gets much higher levels of non-encapsulated (free) MPS than of MPS derived from nSSL-MPS. This suggests much less GC first-pass metabolism, which is relevant to GC side effects [2]. At 1 hour post injection the MPS ratio of brain to liver distribution is 12 times higher than at 0.3 hour for free MPS (compare 0.006 for free with 0.076 for nSSL-MPS). At 24h post injection only MPS derived from nSSL is found in the brain at a brain to liver ratio of 0.18.

**Table 1:** Accumulation of MPS originating from nSSL-MPS and of free MPS in the liver and brain of acute EAE-induced mouse.

Formulation	Time (h)	Liver^a^	Brain^a^	Brain/liver ratio
Free MPS	0.3	112.30	0.36	0.003
Free MPS	24	1.10	0.00	0.000
nSSL-MPS	1	48.82	3.65	0.075
nSSL-MPS	24	3.01	0.54	0.180

a —µg MPS per gram organ.

Comparison of pharmacokinetics and biodistribution of nSSL-MPS in healthy and EAE mice show lack of major differences (data not shown), with the brain being an exception: the concentration of MPS at 1 and 6 h post IV injection in the diseased EAE mice brains was about twice that in the healthy mice **Fig. S1a** (Supporting information)**.** A similar increase in brain nSSL levels was also observed (data not shown) suggesting that most of the drug reached the brain as liposomal drug (which is also obvious from data presented in Table **1**). This is highly significant especially since the brain is a difficult organ to reach by “free” drugs and should be more so for particulates. This enhanced permeability to nano-particulates may be explained by changes in the vascular system that occurs due to the inflammation typical of the EAE brain [36], [37].

#### Proof of controlled zero-order, slow drug release of nSSL in mice

The release rate *in vivo* is calculated from the time-dependent reduction in drug/liposomes (MPS/^3^H-nSSL) ratio in blood and tissues. No reduction in this ratio should occur if there is no drug release, as was well presented by Metselaar et al. [38] for nanoliposomes passively loaded with the water-soluble prodrug prednisolone phosphate. This suggests in the latter case that there is no drug release of the liposomes *in vivo*. The pharmacokinetic and biodistribution studies done with the nSSL-MPS clearly demonstrate *in vivo* zero- order, slow release kinetics in blood (**Fig. 5**) and tissues (**Fig. S1c** Supporting information), which is similar to what was found for these nSSL *in vitro*.

The t_1/2_ of 17 h determined for the release *in vivo* in the bloodstream (**Fig. 5**) although slow, is 3 time faster than the t_1/2_ of 50 h found *in vitro* **(** **Fig. 4a** and inserted **Table**). The reason for the discrepancy between the *in vitro* (in test tube) and *in vivo* (in mice) t_1/2_ is not yet clear. It may be related to the fact that the *in vitro* results were obtained under static conditions at which the volume of the extra-liposome medium is constant, while *in vivo* the free drug once released to the plasma is further cleared to the interstitial fluids of other tissues and organs, which enhances the rate of drug release (this assumption is now under evaluation).

### Demonstrating therapeutic efficacy of nSSL-MPS in animal models

#### Treatment of proteolipid protein (PLP)-induced acute EAE

We also studied therapeutic efficacy in the proteolipid protein (PLP)-induced acute mouse EAE, a routinely used model for the autoimmune inflammatory neurodegenerative disease multiple sclerosis [39], [40], in which the involvement of inflammation in the brain enhance the permeability of this difficult to reach organ.

We tried to utilize previously reported data that showed that nanoliposomes are able to reach the inflamed brain [36], [37]. In this regard Schmidt et al. [36], showed that nSSL passively loaded with the water-soluble GC prodrug prednisolone phosphate have a fivefold better therapeutic efficacy than an equal dose of free prednisolone phosphate. The superiority of the liposomes' based nano-drug is explained in part by accumulation of the nanoliposomes in the diseased CNS. Siegal et al., [41] showed enhanced permeability of Doxil^TM^ in a mouse brain tumor model. Namely, at least in some diseased brains the BBB is partially compromised to the level it enables penetration of nanoliposomes to the diseased tissue.

In this study we also used a clinical multiple sclerosis treatment protocol referred to as “pulse therapy” [42], in which the MPS IV administration is repeated until the autoimmune attack is diminished. Accordingly, we started with IV administration of 50 mg/kg MPS when mild clinical symptoms appeared.

**Figure 6:**
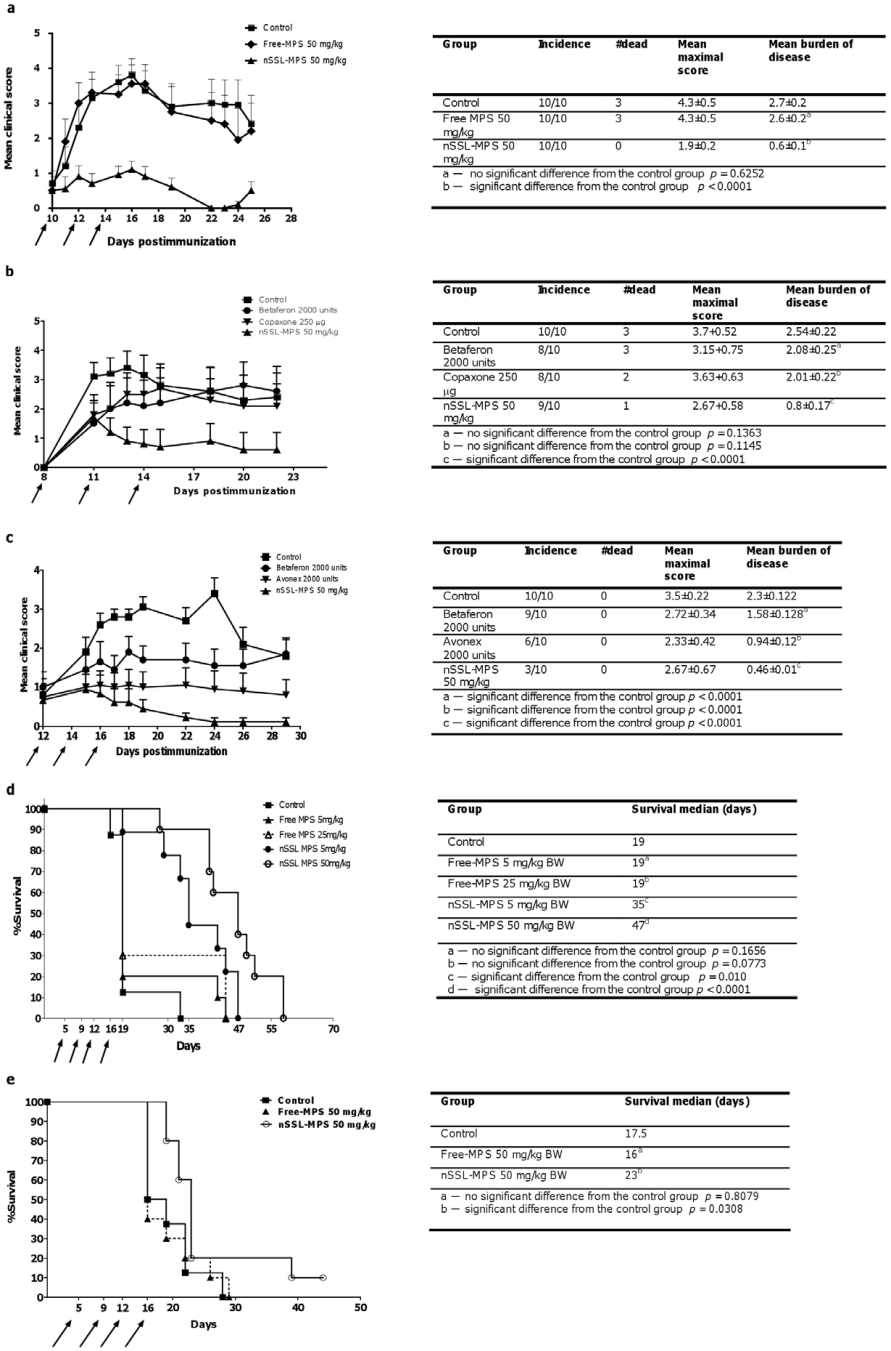
Therapeutic activity of nSSL-MPS in mice induced with EAE (multiple sclerosis model) and in a BCL-1 B-cell leukemia mice animal model (cancer model). (a) Comparison of the therapeutic efficacy of nSSL-MPS and non-liposomal (free) MPS in acute EAE mice model. SJL/J mice (n = 10) were treated by IV injections on days 10, 12, 14 with: nSSL-MPS 50 mg/kg BW (▴), free MPS 50 mg/kg BW (♦), and dextrose 5% (control) (▪). (b) Comparison of therapeutic efficacy of nSSL-MPS with clinically available drugs, Copaxone and Betaferon on acute EAE mouse model. SJL/J mice (n = 10) were treated by IV injections on days 8, 11 and 14 with: nSSL-MPS 50 mg/kg BW (▴); Copaxone 250 µg (▾); Betaferon 2000 units (•); dextrose 5% (control) (▪). (c) Comparison of therapeutic efficacy of nSSL-MPS and two types of interferon- beta on MOG peptide-induced chronic EAE mouse model. C57Bl/6 mice (n = 10) were treated by IV injections on days 12, 14, and 16 with: nSSL-MPS 50 mg/kg BW (▴); Avonex 2000 units (▾); Betaferon 2000 units (•); dextrose 5% (control) (▪). Inset Tables — For all of the above experiments detailed disease-characteristics are given. (d) Survival curve of mice induced with BCL-1 B-cell leukemia. BALB/c mice (n = 10) were IV injected on days 5, 9, 12 and 16 with either free MPS 5 mg/kg BW (▴), nSSL-MPS 5 mg/kg BW (•) free MPS 25 mg/kg BW (Δ dashed line) or nSSL-MPS 50 mg/kg BW (○). The control group (n = 6) did not receive any treatment (▪). (e) Survival curve of mice induced with J6456 mouse T-cell lymphoma cells. BALB/c mice (n = 10) were IV injected on days 5, 9, 12 and 16 with either free MPS 50 mg/kg BW (▴ dotted line) or nSSL-MPS 50 mg/kg BW (○). The control group (n = 6) did not receive any treatment (▪).

**Fig. 6a** and its inserted **Table** demonstrate the superior therapeutic efficacy of nSSL-MPS over free MPS. Firstly, no mortality occurred in the nSSL-MPS group of 10 mice, while in the control and free MPS groups 3 out of 10 mice died. Secondly, looking at the progress of the clinical score, not only was the severity of disease of the nSSL-MPS-treated group much lower, but also recovery from the acute disease attack was much faster. On day 22 mice of the nSSL-MPS treated group reached a clinical score of ∼0.0 (no clinical symptoms) compared with a clinical score of 2.5 for the free-MPS-treated mice, and 3.0 for the control group treated with 5% dextrose.

Thirdly, from the statistical evaluation of the data collected from this experiment, it is obvious that nSSL-MPS are highly efficacious in reducing disease severity compared with the control untreated group, or the group treated with a high dose of free drug [compare the mean maximal score of 1.9 in the nSSL-MPS group (limp tail with righting reflex) with the score of 4.3 (early paralysis) for the group treated with free drug].

#### Comparison between nSSL-MPS and conventional multiple sclerosis drugs in the acute EAE model

We also studied in the same mouse model how the therapeutic efficacy of our nSSL-MPS treatment compares with treatment by the clinically used multiple sclerosis drugs Betaferon and Copaxone.

Betaferon (interferon beta-1b) [43], is a bacterial-derived recombinant cytokine having anti-inflammatory activity. Copaxone [44] (glatiramer acetate) is a polypeptide composed of amino acids that mimic amino acids found in the main myelin-sheath protein. Though the exact therapeutic mechanism of Copaxone is not clear [45], in multiple sclerosis patients it causes decrease in relapses and lesions [46].

**Fig. 6b** and inserted **Table** demonstrate that Copaxone and Betaferon exhibit a mild therapeutic efficacy, lowering the mean burden of disease from 2.54±0.22 to 2.08±0.25 and 2.01±0.22, respectively, while nSSL-MPS, which lower the mean burden to 0.8±0.17, was clearly superior.

#### Therapeutic effect of nSSL-MPS in chronic myelin oligodendrocyte glycoprotein (MOG) 35–55 peptide-induced EAE model and its comparison to other clinically available drugs for multiple sclerosis treatment

The chronic MOG 35–55 peptide-induced EAE mouse model serves as another common disease model for multiple sclerosis. Using this mouse model, we compared the therapeutic efficacy of nSSL-MPS to two types of interferon-beta (interferon-beta 1b referred to as Betaferon, and interferon-beta 1a referred to as Avonex.

**Fig. 6c** and inserted **Table** demonstrate that the chronic EAE induced by the MOG protein, which is characterized by milder clinical features than the PLP-induced EAE (as seen by the low mean clinical score and lack of mortality for all groups), is also highly responsive to nSSL-MPS treatment.

A comparison with Betaferon and Avonex shows clear superiority of the nSSL. While nSSL-MPS is characterized by a mean burden of disease of 0.46±0.01, Betaferon and Avonex are characterized by a mean burden of disease of 1.58±0.13 and 0.94±0.12, respectively. In addition, in terms of recorded disease incidence, nSSL-MPS was also superior, with only 3 incidences, compared to 9 and 6 for the Betaferon and Avonex groups, respectively.

Overall, when we summarize the efficacy of the treatment in the EAE mouse model used by the parameter of “mean burden of disease”, free-MPS has almost no significant effect, while nSSL-MPS was able to lower the mean burden by 4-fold (**Fig. 6a**). These results suggest that only nSSL-MPS, but not standard (non-liposomal) MPS acts as a disease-modifying drug. In addition, nSSL-MPS was more efficacious than Copaxone or interferon-beta 1a/b (**Fig. 6b, 6c**)

#### Treatment of GC-sensitive mouse BCL-1/B-cell leukemia

GCs are also frequently used in cancer treatments targeting various aspects of the disease. These include chemotherapy, complementary treatment to chemotherapy, and supportive palliative treatment for side effects induced by chemotherapy sessions, such as severe edema generated by tumors [47]. This type of treatment is applied to many tumor types irrespective of whether the tumor cells are sensitive or insensitive to the steroids. Recently, Storm and coworkers [15], [48] described a successful treatment of B6F10 murine melanoma by nano-liposomes passively loaded with the GC water-soluble prodrug prednisolone phosphate, where the underlying mechanism was described as inhibition of angiogenesis [15]. However, a greater therapeutic benefit of GCs is their direct ability to ameliorate hematological neoplasms by the mechanism of apoptosis induction [1], which is related to the presence of additional (to the nuclear receptor) mitochondrial GC receptors in these tumors [49] . Some relevant human hematological cancers that involve treatment with GCs are acute lymphoblastic leukemia, chronic myeloid leukemia, Hodgkin's lymphoma, non-Hodgkin's lymphoma, and multiple myeloma [50].

The current treatment protocols of GCs for hematological cancers are based on the administration of high GC doses [51]. This is done in order to satisfy the need to completely obliterate the malignant cells, and to reduce the risk of generating resistance. However, the unfavorable pharmacokinetics and biodistribution of GCs are major obstacles to achieving efficacious and nontoxic treatment. Treatment based on use of nSSL-GC may overcome these major deficiencies, as the same PK and BD advantages that were demonstrated in the EAE mouse model should also apply to the cancers. More importantly, unlike other liposomal GC formulations, which also improve the pharmacokinetic profile of GCs, our liposomal formulation has the unique characteristic of a slow zero-order drug release profile during systemic circulation, making the GC bio-available not only at the inflamed and/or tumor tissue [52], but also to the malignant circulating cells, which will have a much prolonged exposure to the released GC.

This assumption was indeed proven in a BCL-1 B-cell leukemia mouse model [53] where the efficacy of nSSL-MPS was compared to non-encapsulated free-MPS. The BCL-1 cell line [53] is characterized by its high sensitivity to GCs, having an *in vitro* growth inhibition (GI_50_) in the nanomolar range (∼4.2 nM).

**Fig. 6d** shows that while free-MPS at the two doses used (5 mg/kg BW and 25 mg/kg BW) had almost no therapeutic effect (survival median of 19 days), the two doses of nSSL-MPS substantially increased median mice survival (35 days for 5 mg/kg BW and 47 days for 50 mg/kg BW).

#### Treatment of non-GC-sensitive J6456 mouse T-cell lymphoma

In addition to the GC-sensitive experiment described above, we also tested our nSSL-MPS on a non-GC-sensitive lymphoma model in order to check whether this formulation could induce a therapeutic effect that is not the result of a direct cytotoxicity effect, but rather the result of an anti-inflammatory effect. For this we used the J6456 mouse T-cell lymphoma model, which we found to have a GI_50_ value in the mM range (∼1.13 mM). The survival experiment done (**Fig. 6e**) shows that, indeed, the nSSL-MPS had a significant therapeutic effect, where survival median of the J6456 mouse T-cell lymphoma-bearing mice increased from 17.5 to 23 days, while free-MPS treatment had no effect at all (survival median of 16.5 days).

#### Using nSSL-MPS for tumor treatment

The implication of treatment of MPS-sensitive and MPS-insensitive mice tumors for human cancer therapy is that the nSSL-GC may increase the therapeutic index of GCs in both tumor types, with larger direct impact for MPS-sensitive tumors. This can have a major impact on current treatment protocols that combine various chemotherapeutic drugs [50], [51]. Namely, by increasing the therapeutic index of GCs we might lower the dosage of other drugs in the treatment protocol and by that lower the overall toxic effect and perhaps increase the overall therapeutic success of the treatment regime.

### Relevancy of nSSL-MPS nano-drug prototype features to its performance: Conclusions

The optimal nSSL-MPS prototype developed has the following features:

1. Liposome (nSSL) membrane lipid composition determines and controls to a large extent the stability and kinetics (order and rate) of GC release. This is supported by the release activation energy (*E*_a_) calculated from the Arrhenius plot of the process. A high (37.8 kcal/mole) activation energy for nSSL based on high-T_m_ PCs (HSPC and DSPC) supports slow, but sufficient for activity, release at 37°C, concomitantly with minimal release at 4°C (important in order to achieve an acceptable product shelf life).
2. MPS is released from the nSSL-MPS due to its amphipathicity and not due to a “dumping” effect related to damaged or permeable liposome membrane.
3. MPS release profile resembles that of a few other remotely loaded substances such as Doxil^TM^, vincristine, and topotecan [16]–[19], [54].
4. Bulk medium components have only a minimal effect on release kinetics.
5. Overall, the features of the optimized nSSL-MPS described here result in a high drug to lipid ratio (high MPS level per liposome), high efficiency of encapsulation, good stability during 4°C storage, superior pharmacokinetic and biodistribution, as well as a slow zero-order drug release *in vivo*. All these lead to superior therapeutic efficacy in mouse and rat models of diseases with inflammatory components (rheumatoid arthritis [27], EAE (this study), and cerebral malaria (Waknine-Grinberg et al., in preparation) and in two types of mouse tumor models (the MPS-sensitive BCL-1 B-cell leukemia and the MPS-insensitive J6456 T-cell lymphoma).

## Materials and Methods

### Materials

Hydrogenated soybean phosphatidylcholine (HSPC) was obtained from Lipoid KG (Ludwigshafen, Germany). The HSPC has an iodine value of 3.0, and acyl chain composition of 86% stearic acid (C18:0) and 13% palmitic acid (C16:0), and less than 1% of other acyl chains. Its solid-ordered (SO) to liquid-disordered (LD) phase transition occurs at the temperature of maximum change in the heat capacity (T_m_), 52.5°C [35].1-Palmitoyl-2-oleoyl-*sn*-glycero-3-phosphocholine (POPC) and 1,2-distearoyl-*sn*-glycero-3-phosphocholine (DSPC) were obtained from Avanti Polar Lipids, Alabaster, AL, USA. Cholesterol (CHOL) (>99% pure) was obtained from Sigma (St. Louis, MO, USA). N-(carbonyl-methoxypolyethyleneglycol-2000)-1,2-distearoyl-*sn*-glycero-3-phosphoethanolamine, sodium salt (abbreviated as 2000-PEG-DSPE, or PEG-DSPE) was obtained from Genzyme Pharmaceuticals, Liestal, Switzerland, and [^3^H]-cholesteryl hexadecyl ether, was obtained from PerkinElmer, Waltham, MA, USA. EAE immunizing reagents: heat-killed and dried *Mycobacterium tuberculosis* was obtained from Sigma and *Bordetella pertussis* toxin, from Difco.

Glucocorticoid prodrugs: methylprednisolone succinate sodium salt (MPS) was obtained from Pfizer (Puurs, Belgium). Dexamethasone phosphate sodium salt (DEXP), betamethasone phosphate sodium salt (BMP), and hydrocortisone succinate sodium salt (HCS) were obtained from Sigma. Betamethasone hemisuccinate (BMS) was obtained from Steraloids (Newport, RI, USA).

Copaxone® was obtained from Teva, Inc. (Petach Tikva, Israel). Betaferon® (interferon beta-1b) was obtained from Schering-Plough, Inc. (Kenilworth, NJ, USA). Avonex® (interferon beta-1a) was obtained from Biogen, Inc. (Cambridge, MA, USA). Highly pure sterile and pyrogen-free water, of 18.2 ohms resistance, with very low content of total carbon and inorganic ions, was obtained using a WaterPro PS HPLC/Ultrafilter Hybrid model, (Labconco, Kansas City, Mo, USA). This water is referred to as “highly pure water”. All the other chemicals, including buffers, were of analytical grade or better.

### Animals

6–7-week-old female SJL/J, C57Bl/6 and BALB/c mice were obtained from Harlan Laboratories, Jerusalem, Israel.

### Ethical Treatment of Animals

The animals were kept at the Hebrew University Animal Facility and given food and water ad libitum. Experimental use of animals was authorized by the Ethics Committee of the Hebrew University Medical School.

### Methods

#### Surface activity and critical aggregation concentration (CAC) of “water-soluble” GCs

The surface activity and amphipathicity of the “water-soluble” GCs was determined from their effect on aqueous phase surface tension [30], [55]. For this, we measured and compared surface tension of aqueous solutions of the two GCs (MPS and DEXP) over a broad range of concentrations using a µtrougeS tensitometer (Kibron, Helsinki, Finland). Measurements were done using 300 µL aliquots of each GC solution (all pH 7.4) after calibration and zeroing of the sensor using highly purified water having a surface tension of 72 dynes/cm.

Using this method, one can assess if the analyte self-aggregates. The CAC of such an association is determined from the curve describing surface tension as a function of GC concentration [29]. For amphiphiles that self-associate, this curve is biphasic, and the intercept between the two slopes describes the CAC (or critical micelle concentration, CMC, of micelles) [29], [55].

#### Determination of octanol/buffer distribution coefficient

Amphipathicity is commonly described by the analyte octanol/aqueous buffer distribution (D) coefficient test (described by logD) as a function of the aqueous buffer pH.

MPS and DEXP octanol/aqueous buffer logD's were determined by the “shake flask” method [56]. Briefly, to a test tube containing a solution of 1-octanol/HEPES buffer (1/1 v/v), MPS or DEXP was added, and the test tube was shaken vigorously for 1 h; then the test tube was centrifuged to separate the octanol (upper phase) and the buffer (lower phase), followed by determination of MPS or DEXP concentration in each of the phases. LogD was calculated as the log of the ratio of the GC concentration in octanol to that in the buffer.

#### X-ray diffraction analysis of calcium-MPS powder

X-ray diffraction (XRD) analysis was performed on MPS powder, calcium acetate powder, and Ca-MPS powder. A precipitate of Ca-MPS was obtained by mixing 200 mM calcium acetate with 20 mM MPS, followed by centrifugation (7000*g* for 5 min); then the upper phase was discarded and the precipitate was dried at room temperature. XRD powder measurements were performed on a D8 Advance diffractometer (Bruker AXS, Karlsruhe, Germany) with a goniometer radius 217.5 mm, Göbel mirror parallel-beam optics, 2° Sollers slits, and 0.2 mm receiving slit. Using CuKα radiation (λ = 1.5418 Å), XRD patterns were recorded from 2° to 52° 2θ (at room temperature) with the following measurement conditions: tube voltage of 40 kV, tube current of 40 mA, step scan mode with a step size 0.02° 2θ, and counting time of 1s/step. XRD patterns were analyzed using DiffracPlus software.

#### Preparation of liposomes exhibiting intraliposome high/medium low transmembrane calcium acetate gradient

All lipid compositions used for the fabrication of nSSL were lyophilized, then hydrated in the desired acetate salt (usually 200 mM calcium acetate). The fabrication procedure was based on the approach of Peleg-Shulman et al [57]. Extrusion steps were performed above the SO-to-LD phase transition temperature of the liposome-forming PC (>62°C for HSPC or DSPC-based nSSL and room temperature for POPC-based nanoliposomes). Intraliposomal salt gradient (i.e., calcium acetate) was created by dialyzing the nSSL against 5% dextrose in a few steps overnight at 4°C (4 exchanges including final overnight stage) leading to a transmembrane gradient >800. Our final nSSL-MPS are small (mean size of 83±15 nm SD) unilamellar vesicles in 5% dextrose, having a unimodal narrow size distribution and low negative charge (zeta potential of -5mV), which is related to the hindered charge of pegylated liposomes [58].

#### nSSL-MPS chemical and physicochemical characterization

The concentration of MPS and its hydrolysis products were quantified using HPLC [59]. nSSL phospholipid (PL) concentration was quantified using the modified Bartlett procedure [60]. The concentration of calcium (determined by atomic absorption spectrometry (AAS)) was used for determination of nSSL trapped (aqueous) volume. Acetate concentration was determined by a commercial enzymatic kit (Megazyme International, Wicklow, Ireland), which we modified to determine total extraliposome medium, and intraliposome aqueous phase acetate concentrations.

The mean size distributions of nSSL and of nSSL-MPS were determined by a particle sizer ALV-NIBS/HPPS with ALV-5000/EPP multiple digital correlator (ALV-Laser Vertriebsgesellschaft GmbH, Langen, Germany). Zeta potential was determined using Zetasizer Nano Series ZEN2600F (Malvern Instruments, Malvern, UK)

#### Cryo-TEM images and X-ray diffraction studies

Cryo-TEM studies were performed as described in Schroeder et al [31].

#### Preparation of nSSL passively loaded with MPS

nSSL into which MPS was passively loaded were prepared as described above, except that lipid hydration buffer was composed of 100 mg/mL (201 mM) MPS dissolved in 0.9% NaCl.

#### Remote loading of other water-soluble GC prodrugs

The following GC prodrugs were remotely loaded into nSSL exhibiting a transmembrane calcium acetate gradient: hydrocortisone succinate sodium salt (HDS), betamethasone hemisuccinate (BMS), and betamethasone phosphate sodium salt (BMP). Remote loading for all GCs was done as described in Avnir et al [27]. Briefly; GC of choice was dissolved in 5% dextrose and was added to the nSSL dispersion. The nSSL-GC mixture was incubated for 20 minutes at 60–65°C (above the 52.5°C T_m_ of hydrogenated soybean phosphatidylcholine), after which the nSSL-GC mixture was dialyzed against 5% dextrose at 4°C for overnight.

#### Release kinetics of MPS at 37°C from nSSL-MPS in human plasma and PBS buffer

nSSL-MPS samples composed of various defined lipid compositions were incubated at 37°C in either 80% human plasma (60 µl nSSL-MPS / 240 µl human plasma) or in phosphate buffered saline (PBS, pH 7.4) for periods up to 96 h. Aliquots were taken from these samples at the desired time points and analyzed for level of drug released by gel permeation chromatography, using a Sepharose CL-4B column which separates well nSSL from non-liposomal (free) MPS. The columns were equilibrated with 0.9% NaCl and then an aliquot of nSSL-MPS in the desired medium was loaded on the columns; elution was done using 0.9% NaCl, and fractions of ∼1 mL were collected using a fraction collector. Distribution of liposomes in the different fractions collected was determined using the fluorescent probe diphenylhexatriene (DPH), which fluoresces only when present in hydrophobic milieu [61], as described by London and Feligenson [62]. Fractions 4–7, which contained the nSSL, were analyzed for PL concentration, particle size distribution, MPS, MPS derivatives, and Ca^2+^ concentration. Fractions 9–16, which lack nSSL and contain only low molecular weight components, were analyzed for MPS and MPS derivatives. The nSSL analysis of size distribution, when combined with [Ca^2+^]/[PL], mole ratio was used to assess physical integrity of nSSL. The change in [MPS]/[PL] was used to determine kinetics of MPS release from the nSSL.

#### Induction of EAE

The following EAE animal models were used:

Acute EAE — immunization with proteolipid protein [40], which induces a disease characterized by severe clinical symptoms, with no relapsing sessions, and Chronic EAE — immunization with the myelin oligodendrocyte glycoprotein [63], which induces a milder disease, with relapsing sessions.

**1.** Acute EAE was induced in 6–7-week-old SJL/J female mice by subcutaneous injection in the flank of complete Freund's adjuvant containing 150 µg of proteolipid protein (PLP) 139–151 peptide, and 200 µg of *Mycobacterium tuberculosis*. In order to boost the immune system, 150 ng of *Bordetella pertussis* toxin was injected intraperitoneally (IP) immediately and 48 h later. In this EAE model, clinical signs start to appear on days 8–11 post immunization, and are maximal at about day 19.

**2.** Chronic EAE was induced in 6–7-week-old C57Bl/6 female mice by immunizing in the flank on days 0 and 7 with subcutaneous injection of complete Freund's adjuvant containing 300 µg of mouse myelin oligodendrocyte glycoprotein (MOG) 35–55 peptide and 200 µg of *Mycobacterium tuberculosis*. In order to boost the immune system, 500 ng of *Bordetella pertussis* toxin was injected IP immediately and 48 h later. In this EAE model, clinical signs also start to appear on days 8–11 post immunization.

#### Clinical scoring

The animals were monitored for clinical signs and were scored according to the following parameters: 0—Normal behavior, 1—Distal limp tail, 1.5—Complete limp tail, 2—Complete limp tail with righting reflex, 3—Ataxia, 4 —Early paralysis, 5—Full paralysis, 6—Moribund/death. During the experiment, animals that exhibited clinical symptoms above ataxia were given saline as hydration fluid.

In **Fig. 6** for each group the mean daily clinical score is given, and in the inserted Tables, from the combined data the following statistical parameters (using Prism 4 software, GraphPad software, San Diego, CA, USA) are given: mean maximal score, and mean burden of disease (the mean of all the scores throughout all the days of the experiment). For mean burden of disease, a Student's *t* test was used to determine statistical significance at *p*<0.05 by using Prism 4 software.

#### Induction of BCL-1 mouse B-cell lymphoid leukemia

BCL-1 B-cell lymphoid leukemia was induced [53] in 6–7-week-old BALB/c mice by IP injection of 1 million BCL-1 lymphoma cells.

#### Induction of J6456 mouse T-cell lymphoma

J6456 mouse T-cell lymphoma was induced [64] in 6–7-week-old BALB/c mice by IP injection of 1 million J6456 T-cell lymphoma cells.

#### Pharmacokinetics and biodistribution

SJL/J mice induced with acute EAE and control mice were injected on day 11 with either free MPS (50 mg/kg BW), or ^3^H-CE labeled-nSSL-MPS (50 mg/kg of MPS). At 1, 6, 24, and 48 h sera were collected, and then for each time point, animals (n = 3) were sacrificed to harvest their organs.

Organs were homogenized (Polytron, Kinematica GmbH, Germany), and then MPS and its derivatives were extracted according to Smith (1979) [65]. Radiolabeled samples were tested for level of liposomal marker (^3^H-cholesteryl hexadecyl ether ^3^H-CE) in homogenates using a sample oxidizer (Model 307, Packard Instrument Co., Meriden, CT, USA).

## Supporting Information

Scheme S1 **Principles and mechanism of nSSL transmembrane calcium acetate gradient-driven remote loading and release mechanisms of amphipathic weak acids such as MPS.** (a) Fabrication stage of nSSL having transmembrane calcium acetate gradient. Calcium acetate at the desired concentration is passively loaded into intraliposome aqueous phase during lipid hydration to form multilamellar liposomes. This is followed by extrusion to form nSSL. Calcium acetate is removed from the extraliposome medium by repeated dialysis resulting in intraliposome high/extraliposome-low calcium acetate gradient. In the intraliposome aqueous phase: 1. Calcium acetate is dissociated to calcium cations and acetate anions in a pH and concentration- dependent manner. The intraliposome Ca^2+^ ion concentration in the intraliposome aqueous phase is calculated as {[Ca^2+^]/[PL]} /[trapped volume (µl) / [PL} Ca^2+^ and PL were determined as described in Methods. Trapped volume for more than ten nSSL-MPS batches was 1.15±0.11 µl/µmole PL and [Ca]/[PL 0.38-to-0.28 mM/mM. This results in an intraliposome Ca^2+^ concentration of ∼200 mM and a gradient of calcium acetate before loading of >800 (Turgeman et al., in preparation). 2. The nonprotonated charged acetate is in pH-dependent equilibrium with the uncharged protonated acetic acid (p*K*_a_  = 4.75). 3. Acetic acid (but neither acetate nor calcium ions) can diffuse across the liposome membrane to the external medium. Indeed, Ca^2+^ ion concentration is unaffected by the MPS loading. 4. The release of acetic acid induces pH increase in the intraliposome aqueous phase to the extent that the equilibrium of acetate shifts the reaction to the direction of the nonprotonated, charged acetate anion, and therefore release of acetic acid is slowed down or practically stops. The pH changes were measured using pyranine after correction for the presence of Ca^2+^ ions [66]. **(b)** Stage of remote loading of amphipathic weak acid GC prodrugs into nSSL. 1. The products of (**a**) (above) are nano-liposomes that have lost a small fraction of their acetate so they have a small excess of calcium cations balanced electrostatically by hydroxyl anions, which shifts the intraliposome pH to be slightly alkaline. Therefore, these preformed liposomes are “eager” to “pump in” the amphipathic weak acid GC (drug-D) from the extraliposome medium. 2. When an amphipathic weak acid GC prodrug (i.e., MPS) is added to the extraliposome aqueous medium, it diffuses into the intraliposome aqueous phase in its protonated (un-ionized) form. In the alkaline intraliposome aqueous phase (see **b1**) it undergoes deprotonation (ionization). 3. The deprotonated (ionized DCOO^-^) of the weak acid drug, will form a salt with Ca^2+^ [Ca(MPS)_2_], which precipitates due to its high concentration and low solubility in the nano-aqueous volume of the nano-liposome. 4. These two processes, the deprotonation of the amphipathic weak acid GC and the concomitant formation of a calcium salt, lead to lowering of the intraliposomal pH, thereby reducing the intraliposome pH. This translates into favorable conditions for the protonation of more acetic acid, which induces is the repeating and recycling the conditions described in part **a2-to-a4** of this scheme. This “pumping” process can continue until all acetate ions are exchanged with the loaded amphipathic weak acid (MPS). To ensure loading stability, the process is stopped before the entire acetate gradient is utilized. All these steps are well documented in the Results and Discussion section above.
(TIF)

Figure S1 **Time-dependent tissue biodistribution of MPS originating from nSSL-MPS. (a)** The time-dependent biodistribution of MPS originating from IV administered nSSL-MPS is shown as a ratio of percent injected dose per gram tissue for the indicated organs measured at 1, 6, 24, and 48 h post IV injection. **(b).** Time-dependent biodistribution of ^3^H-CE originating from ^3^H-nSSL-MPS is shown as a ratio of percent injected dose per gram tissue for the indicated organs measured at 1, 6, 24, and 48 h post IV injection. **(c)** Profile of MPS release from ^3^H-nSSL-MPS in selected organs presented as the time-dependent ratio [% injected dose MPS^ (of nSSL-MPS)^] / [% injected dose ^3^H-CE^ (of nSSL-MPS)^] at 1, 6, and 24 h post IV injection.
(TIF)
