# Supplementary figures and images for: Fabrication Principles and Their Contribution to the Superior In Vivo Therapeutic Efficacy of Nano-Liposomes Remote Loaded with Glucocorticoids
Source: PLoS One. 2011 Oct 6;6(10):e25721. doi: 10.1371/journal.pone.0025721 (PMC3188566; doi:10.1371/journal.pone.0025721)

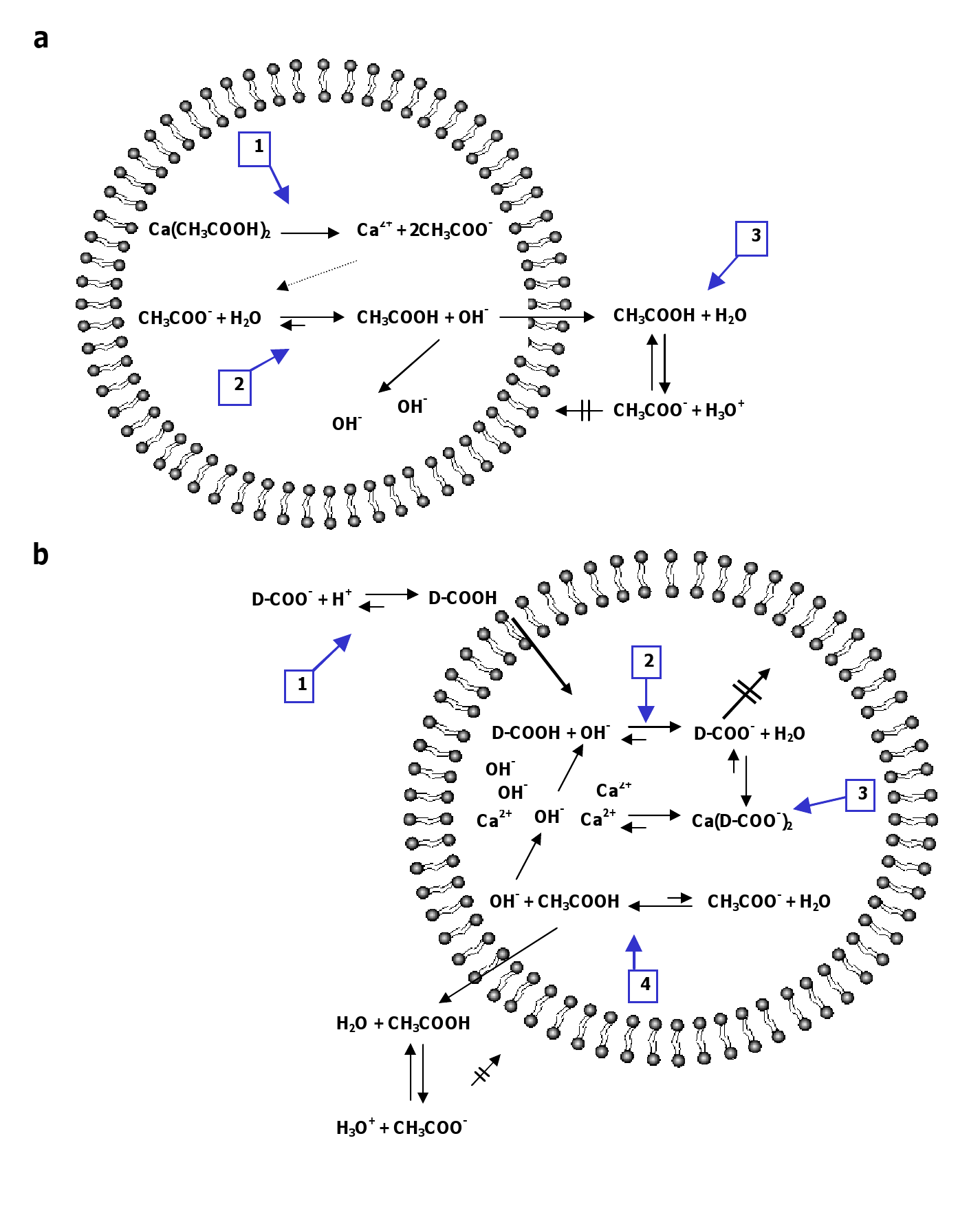

Supplement: Scheme S1 — Principles and mechanism of nSSL transmembrane calcium acetate gradient-driven remote loading and release mechanisms of amphipathic weak acids such as MPS. (a) Fabrication stage of nSSL having transmembrane calcium acetate gradient. Calcium acetate at the desired concentration is passively loaded into intraliposome aqueous phase during lipid hydration to form multilamellar liposomes. This is followed by extrusion to form nSSL. Calcium acetate is removed from the extraliposome medium by repeated dialysis resulting in intraliposome high/extraliposome-low calcium acetate gradient. In the intraliposome aqueous phase: 1. Calcium acetate is dissociated to calcium cations and acetate anions in a pH and concentration- dependent manner. The intraliposome Ca2+ ion concentration in the intraliposome aqueous phase is calculated as {[Ca2+]/[PL]} /[trapped volume (µl) / [PL} Ca2+ and PL were determined as described in Methods. Trapped volume for more than ten nSSL-MPS batches was 1.15±0.11 µl/µmole PL and [Ca]/[PL 0.38-to-0.28 mM/mM. This results in an intraliposome Ca2+ concentration of ∼200 mM and a gradient of calcium acetate before loading of >800 (Turgeman et al., in preparation). 2. The nonprotonated charged acetate is in pH-dependent equilibrium with the uncharged protonated acetic acid (pK a = 4.75). 3. Acetic acid (but neither acetate nor calcium ions) can diffuse across the liposome membrane to the external medium. Indeed, Ca2+ ion concentration is unaffected by the MPS loading. 4. The release of acetic acid induces pH increase in the intraliposome aqueous phase to the extent that the equilibrium of acetate shifts the reaction to the direction of the nonprotonated, charged acetate anion, and therefore release of acetic acid is slowed down or practically stops. The pH changes were measured using pyranine after correction for the presence of Ca2+ ions [66]. (b) Stage of remote loading of amphipathic weak acid GC prodrugs into nSSL. 1. The products of (a) (above) are nano- [file pone.0025721.s001.tif]

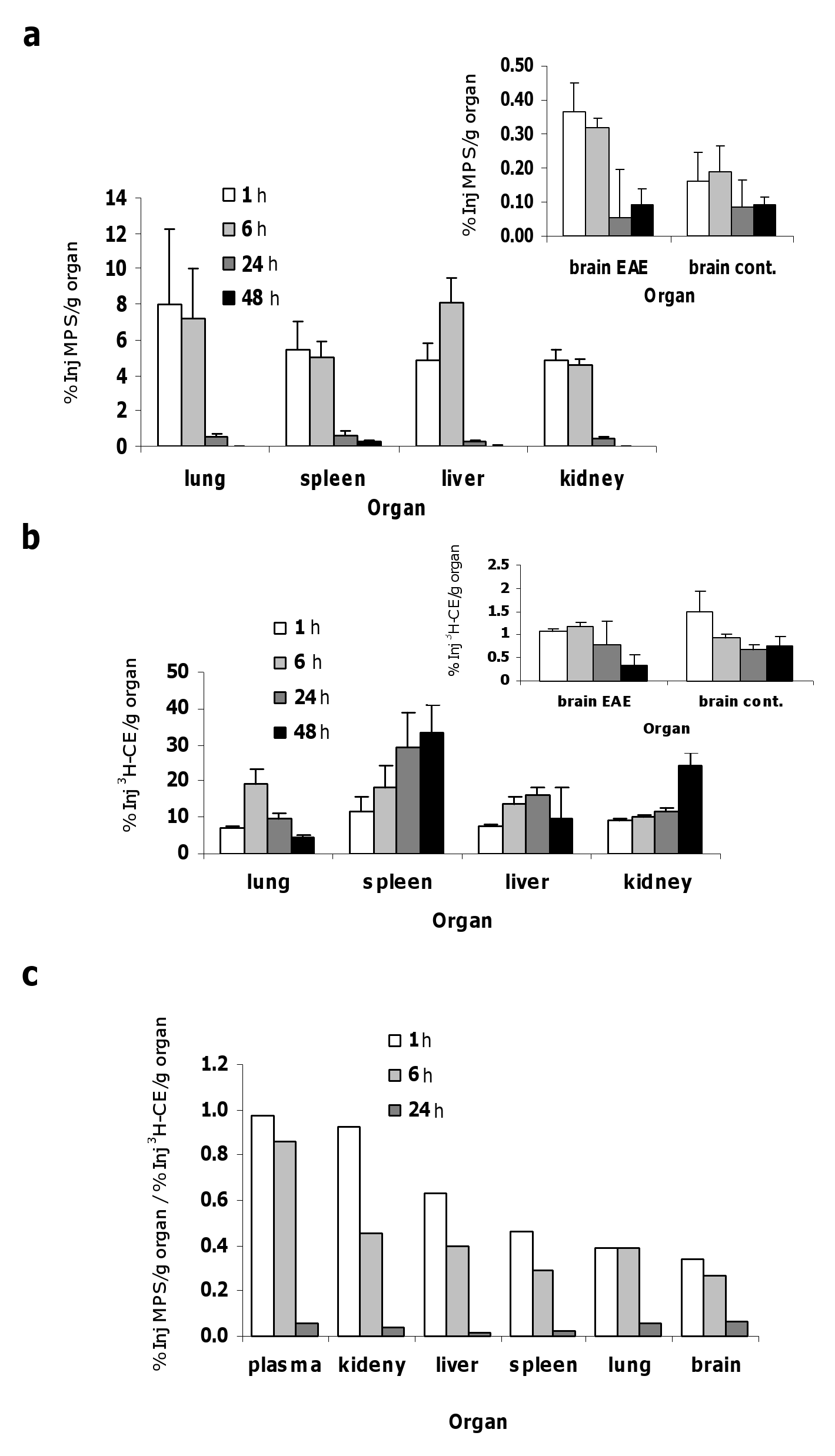

Supplement: Figure S1 — Time-dependent tissue biodistribution of MPS originating from nSSL-MPS. (a) The time-dependent biodistribution of MPS originating from IV administered nSSL-MPS is shown as a ratio of percent injected dose per gram tissue for the indicated organs measured at 1, 6, 24, and 48 h post IV injection. (b). Time-dependent biodistribution of 3H-CE originating from 3H-nSSL-MPS is shown as a ratio of percent injected dose per gram tissue for the indicated organs measured at 1, 6, 24, and 48 h post IV injection. (c) Profile of MPS release from 3H-nSSL-MPS in selected organs presented as the time-dependent ratio [% injected dose MPS (of nSSL-MPS)] / [% injected dose 3H-CE (of nSSL-MPS)] at 1, 6, and 24 h post IV injection. (TIF) [file pone.0025721.s002.tif]
